# Association of serum sestrin 2 and betatrophin with serum neutrophil gelatinase associated lipocalin levels in type 2 diabetic patients with diabetic nephropathy

**DOI:** 10.1007/s40200-020-00498-0

**Published:** 2020-02-06

**Authors:** Khalid M. Mohany, Osamah Al Rugaie

**Affiliations:** 1 Medical Biochemistry Department, College of Medicine, Assiut University|, Asyut, Egypt; 2 Basic Medical Sciences Department, Unaizah College of Medicine, Qassim University, Buraydah, Kingdom of Saudi Arabia

**Keywords:** Serum sestrin2, Serum betatrophin, sNGAL, Diabetic nephropathy

## Abstract

**Purpose:**

Understanding the pathogenesis and the molecular mechanisms of diabetic nephropathy (DN) helps its timely detection and prevention. The current work aims tomeasure serum sestrin 2 and betatrophin levels in healthy and type diabetic (T2DM)subjects with/or without diabetic nephropathy (DN) and also to test their correlation with serum neutrophil gelatinase associated lipocalin (sNGAL); indicator of DN.

**Methods:**

This study included 96 subjects; 20 healthy (G1) and 76 T2DM [22 normoalbuminuric (G2), 35 microalbuminuric (G3) and 19 macroalbuminuric (G4)]. Serum sestrin 2, betatrophin and NGAL were measured by their corresponding kits.

**Results:**

Significant low levels of serum sestrin 2 andhigh levels of serum betatrophin were found in T2DM group when compared to G1 (*p* = 0.002,*p* > 0.001, respectively) and this difference is manifested in G4 followed, in order, by G3, G2 then G1 (*p*= > 0.001 for both). Also, serum sestrin2 levels showed significant negative correlations with sNGAL in G1 (r = −0.497, *p* = 0.026), G2 (r = −0.784, *p* > 0.001), G3 (r = −0.894, *p* > 0.001) and G4 (r = −0.896, pp. > 0.001) while serum betatrophin levels showed significant positive correlations with sNGAL in G2 (r = 0.681, *p* > 0.001), G3 (r = 0.518, *p* > 0.001) and G4 (r = 0.727, *p* > 0.001).

**Conclusion:**

Serum sestrin 2 levels decrease significantly while betatrophin levels increase significantly in T2DM patients with DN especially those with macroalbuminuria. These levels have significant effect strengths on the indicator of diabetic nephropathy; sNGAL which might indicate theirvaluablerole in the timely detection and prevention of the development of DN.

## Introduction

Diabetes mellitus is a syndrome with elevated blood glucose along with abnormal carbohydrate, lipid and protein metabolism [1]. Globally, about 400 million people were estimated to have diabetes in 2010; Type 2 diabetes mellitus (T2DM) constituted more than 90 % of the cases [2]. Diabetes mellitus is accompanied with a high risk of developing microvascular complications such as diabetic nephropathy (DN) [3–5]. The development of DN is associated albuminuria, hypertension, deteriorated functions glomeruli and tubulointerstitial injure [6, 7]. Unless macroalbuminuria occurs, DN is preventable by controlling blood glucose [8] which indicates the importance of understanding the pathogenesis and the timely detection of DN.

Both T2DM and obesity are considered as inflammatory conditions and the control of their accompanied systemic inflammation may improve the both conditions and minimize their complications [9–11]. Sestrin 2 (His 95, 54.5 Kda, 480 aminoacid) belongs to a group of proteins that are induced under stress conditions such as oxidative stress, inflammation and DNA damage. It protects the cell from oxidative damage and helps to maintain normal cell metabolism, homeostasis, growth and survival [12, 13]. A decrease in the intracellular levels of sestrin 2 can lead many adverse sequelae such as oxidative damage, mitochondrial dysfunction, and insulin resistance [14, 15]. The investigation of sestrin 2 levels in obese and/or T2DMpatients revealed controversial results [14, 16, 17].

Neutrophil gelatinase associated lipocalin (NGAL) is a twenty-five Kilo-Dalton protein that helps the transmembrane transportation of several water-insoluble molecules (e.g.siderophores) through areceptor mediated endocytosis [18, 19]. Estimation of serum NGAL (sNGAL) levels were proved to bevital in the prediction and timely detection of renal tubular damage [18, 20]. Also, several studies reported early rises in sNGAL levels in diabetic patients even before renal involvement. In this regard, measurement of sNGAL levels was suggested for prediction and early detection of DN [20, 21].

Betatrophin (MW; 22 KDa) is a hormone that affects the proliferation, growth and mass expansion of beta-cells of Langerhans and enhances glucose tolerance [22]. It also functions to regulate lipid metabolism [23]. Its expression occurs mainly in liver and adipose tissues [22]. Several studies tested the relation between serum betatrophin and glucose levels, insulin resistance and other biomarkers in T2DM;the results were controversial [24–28].

The current work aims to compare serum sestrin 2 and betatrophin levels in T2DM patients (with or without DN) with healthy individuals. Also, to find out the correlation and the effect strength of sestrin 2 and betatrophin levels with/on the levels ofsNGAL. Moreover, to test the efficacies of serum sestrin 2 levels and betatrophin levels in differentiating patient with diabetic nephropathies from diabetic-only individuals, to propose or reject a possible therapeutic use of sestrin 2 or betatrophinin the prevention of DN.

## Subjects and methods

The current study is a case-control that was done in Unaizah college of medicine, Qassim University, Saudi Arabia in collaboration with diabetic outpatient clinic between March and July, 2018after being approved by the Qassim regional committee of bioethics. Written consents were taken from all study participants. The study included 96subjects; 37 men and 59 women, their age was between 35 and 70 years. They were categorized into: G1; control, 20 non-diabetic individuals and cases (76 T2DM patients; treated by life style-modifications and oral hypoglycemic drugs). According to urinary albumintocreatinineratio (ACR), the cases were categorized into 3 groups:G2;22 normoalbuminuric case (ACR >20 mg/g), G3: 35 microalbuminuric case (ACR = 20 to 200 mg/g) and G4: 19 macroalbuminuric case (ACR < 200 mg/g) [29, 30]. Exclusion criteria included subjects with type 1 diabetes mellitus or when the participant presented with any systemic or endocrinal diseases(e.g. infections, hypertension, Cushing syndrome, fever…etc.)

From all eligible subjects, 6 mL blood was drawn from the antecubital vein. Two mL was stored as whole blood till the assay of glycosylated hemoglobin percentage by its corresponding kit. The serum of the other 4 mL was kept in aliquotsat −80 °C till the assay of other parameters. Serum sestrin 2 levels were measured by the sestrin 2 ELISA Kit (Wuhan Fine biotech, Hubei Province, China, Catalogue # ABIN5521614).Serum betatrophin was measured by Human Angiopoietin-like protein 8/Betatrophin ELISA Kit (Novus biologicals, Catalogue # NBP2–68217).Human NGAL/LIPOCALIN-2 ELISA kit was used to measures NGAL (Aviscera Bioscience, Santa Clara, USA, Cat. No: skoo233–02). The other serum parameters were measured by using routine procedures in the chemical laboratory.

The collected data were analyzed by SPSS 25.0 for windows. Two Independent quantitative variables were compared by Student T-test. More than 2 independent quantitative variables were tested by one-way analysis of variance (ANOVA). Correlations between various variables were done by Pearson’s method. Multiple linear regression analysis in the total sample was done to test independent associations between significant variables with sNGAL as dependent variable. *p* Value was considered significant when≤0.05. The receiver operating characteristic (ROC) was done to test the efficacies of serum sestrin 2 and betatrophin levels in differentiating patient with diabetic nephropathies from diabetic-only individuals.

## Results

Significant low levels of serum sestrin 2 were found in T2DM patients when compared to the healthy control group while the levels of serum betatrophin and sNGAL were significantly high (Table 1).

**Table 1:** Mean ± SD of different parameters in the control group and T2DM cases

		Control	T2DM cases	*p* value
		G1	G2, G3 and G4	
		(*n* = 20)	(*n* = 76)	
Age (years)		47.5 ± 9.9	49.5 ± 9.0	0.373
Gender	Male	13 (65%)	24 (31.6%)	0.007
	Female	7 (35%)	52 (68.4%)	
BMI (kg/m^2^)		21.8 ± 3.1	28.3 ± 3.3	>0.001
s-FG (mmol/L)		5.2 ± 0.34	6.8 ± 1.9	>0.001
s–Cr (mg/dL)		1.2 ± 0.19	1.4 ± 0.23	0.008
s- alb (g/L)		40.5 ± 3.2	36.9 ± 4.7	0.002
GA %		13.1 ± 1.6	15.5 ± 4.6	0.026
HbA1c%		6.5 ± 0.9	8.11 ± 2.0	>0.001
Serum sestrin2 (ng/mL)		5.9 ± 1.9	4.1 ± 2.3	0.002
Serum betatrophin (ng/mL)		30.9 ± 5.8	42.3 ± 9.7	>0.001
sNGAL (ng/mL)		66.7 ± 21.6	282.8 ± 198.5	>0.001

*T2DM* type 2 diabetes mellitus, *SD* standard deviation, *BMI* body mass index, *s-FG* serum fasting glucose, *s–Cr* serum creatinine, *s-alb* serum albumin, *GA%* serum glycated albumin%, *HbA1c%* glycosylated hemoglobin%, *sNGAL* serum neutrophil gelatinase associated lipocalin

Sestrin 2 levels were significantly low in G4 followed, in order, by G3, G2 then G1 while serum betatrophin and sNGAL levels were significantly high in G4 followed, in order, by G3, G2 then G1 (Table 2).

**Table 2:** Mean ± SD of different parameters in the 4 studied groups

		Control	T2DM patients			*p* value
		G1 (*n* = 20)	G2 (*n* = 22)	G3 (*n* = 35)	G4 (*n* = 19)	
Age (years)		47.5 ± 9.9	47.0 ± 9.2	48.7 ± 8.5	53.9 ± 8.4	0.066
Gender	Male	13 (65%)	8 (36.4%)	12 (34.3%)	4 (21%)	0.034
	Female	7 (35%)	14 (63.6%)	23 (65.7%)	15 (79%)	
Dur. (years)		–	13.9 ± 6.1	17.7 ± 7.5	18.9 ± 4.8	0.02
BMI (kg/m^2^)		21.8 ± 3.1	27.6 ± 3.3	28.2 ± 3.3	29.1 ± 3.2	>0.001
s-FG (mmol/L)		5.2 ± 0.34	6.3 ± 1.9	6.8 ± 1.8	7.2 ± 2.1	0.002
s–Cr (mg/dL)		1.2 ± 0.19	1.33 ± 0.20	1.3 ± 0.24	1.5 ± 0.20	0.010
s- alb (g/L)		40.5 ± 3.2	38.7 ± 3.6	37.0 ± 4.3	34.9 ± 5.7	0.001
GA %		13.1 ± 1.6	14.2 ± 3.1	15.5 ± 4.7	16.9 ± 5.5	0.024
HbA1c%		6.5 ± 0.9	7.0 ± 2.0	7.6 ± 2.3	8.5 ± 1.6	0.022
Serum sestrin2 (ng/mL)		5.9 ± 1.9	5.6 ± 2.9	3.7 ± 1.7	3.1 ± 1.4	>0.001
Serum betatrophin (ng/mL)		30.9 ± 5.8	34.4 ± 1.9	42.1 ± 8.4	51.7 ± 9.2	>0.001
sNGAL (ng/mL)		66.7 ± 21.6	158.7 ± 83.2	211.3 ± 77.1	558.4 ± 189.5	>0.001

*T2DM* type 2 diabetes mellitus, *SD* standard deviation, *Dur* duration of diabetes in years, *BMI* body mass index, *s-FG* serum fasting glucose, *s–Cr* serum creatinine, *s-alb* serum albumin, *GA%* serum glycated albumin%, *HbA1c%* glycosylated hemoglobin%, *sNGAL* serum neutrophil gelatinase associated lipocalin

In the whole sample, sestrin 2 levels showed significant negative correlations with both serum betatrophin levels (r = − 0.570, *p* > 0.001) and serum NGAL levels (r = − 0.620, *p* > 0.001). Betatrophin serum levels showed a significant positive correlation with serum NGAL levels (r = 0.776, *p* > 0.001). The correlations between serum sestrin 2, betatrophin and NGAL with other studied parameters in the whole sample is shown in Table 3.

**Table 3:** Correlations between serum sestrin 2, betatrophin and NGAL and other studied parameters in the whole sample

		Age (years)	Dur.(years)	BMI (kg/m2)	s-FG(mmol/L)	s–Cr(mg/dL)	s-alb(g/L)	GA %	HbA1c%
Sestrin2 (ng/mL)	r	−0.316	−0.404	−0.415	−0.413	−0.389	−0.367	−0.529	−0.360
Betatrophin (ng/mL)	r	0.403	0.479	0.397	0.532	0.462	−0.394	0.623	0.606
sNGAL (ng/mL)	r	0.464	0.513	0.409	0.476	0.450	−0.397	0.581	0.486

All correlations mentioned in the table are significant at the 0.01 level (2-tailed)

*Dur* duration of diabetes in years, *BMI* body mass index, *s-FG* serum fasting glucose, *s–Cr* serum creatinine, *s-alb* serum albumin, *GA%* serum glycated albumin%, *HbA1c%* glycosylated hemoglobin%, *sNGAL* serum neutrophil gelatinase associated lipocalin

**Fig. 1:**
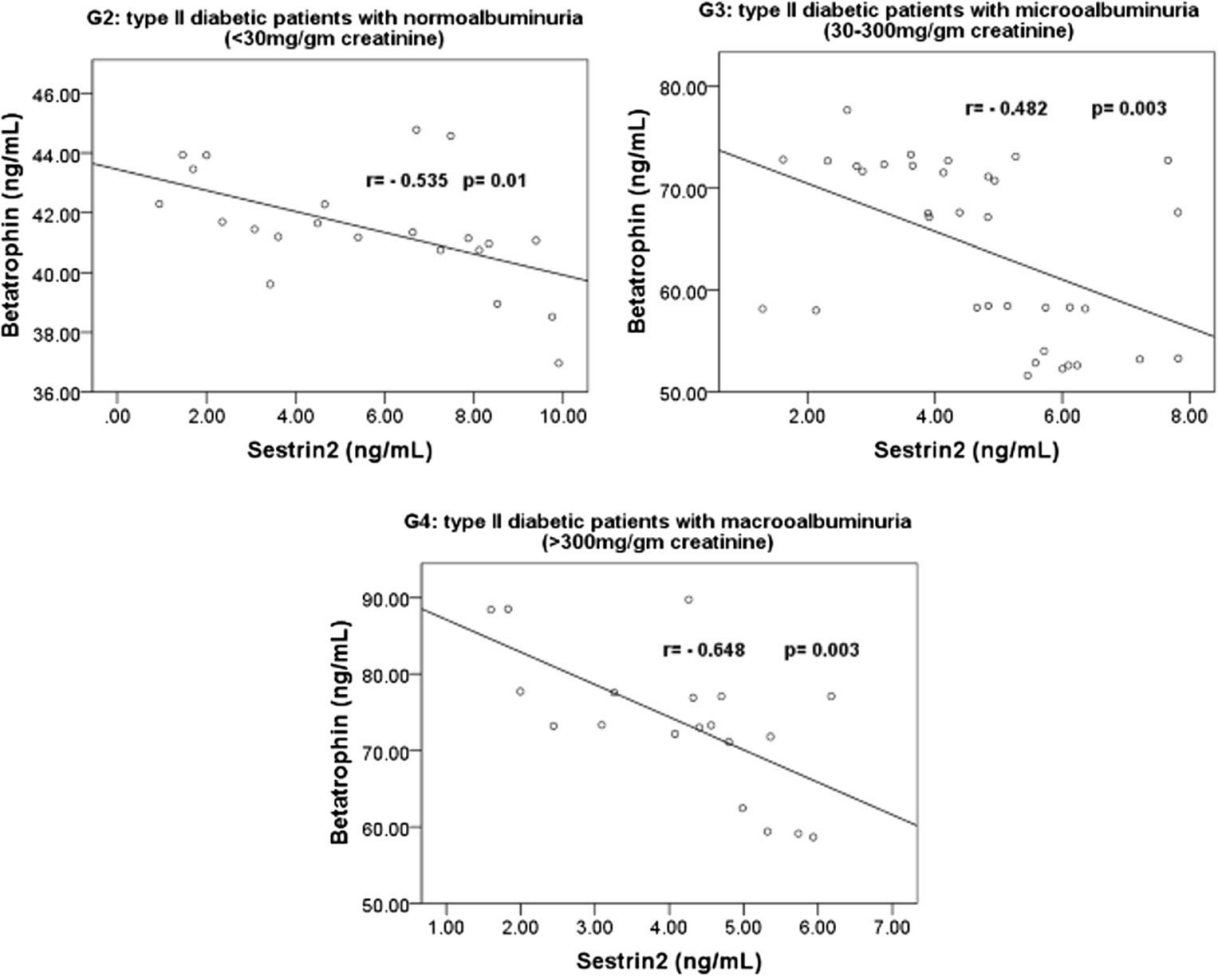
Correlations between serum 2 levels and betatrophin in G2, G3 and G4

**Fig. 2:**
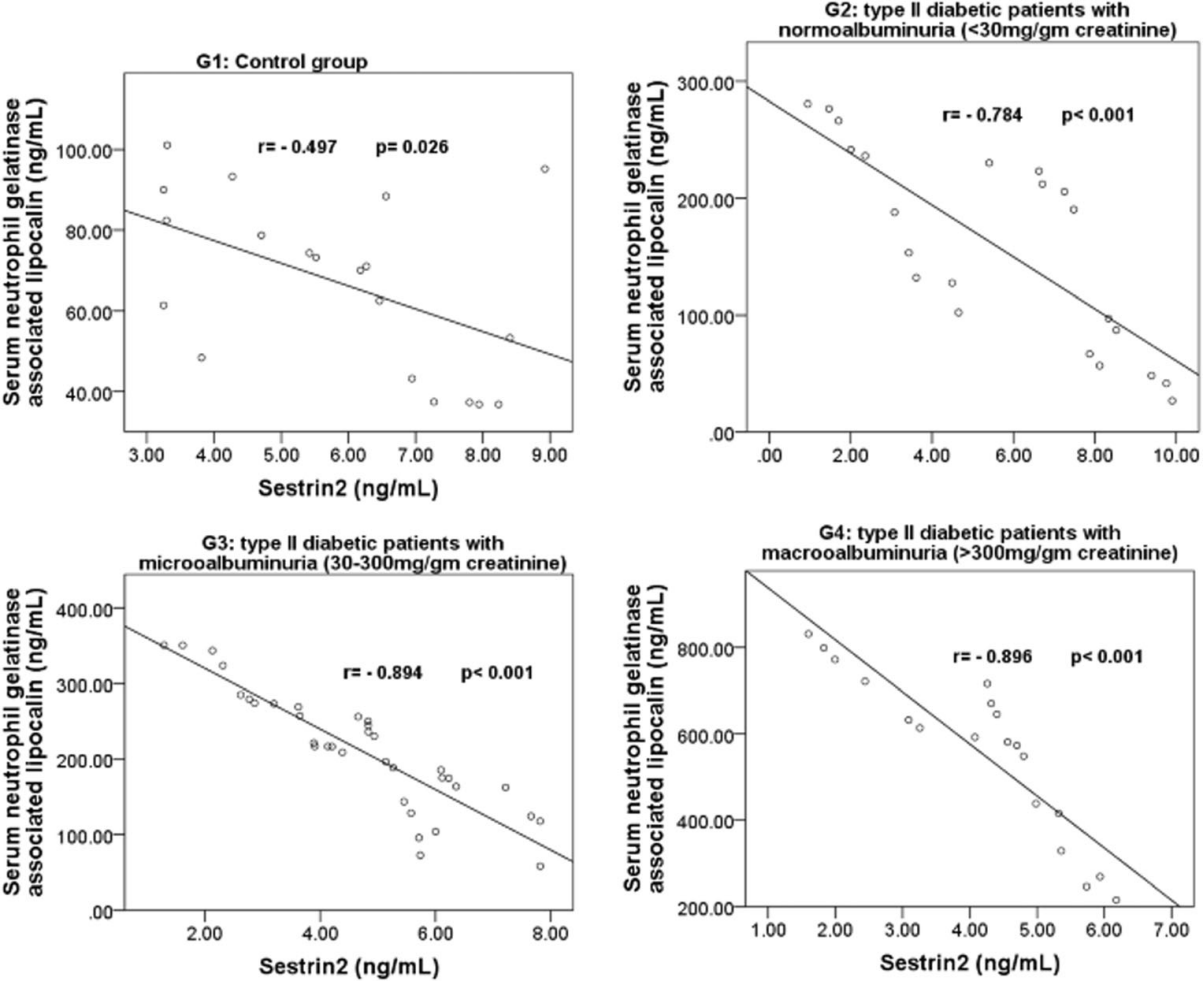
Correlation between serum Sestrin 2 levels and serum neutrophil gelatinase associated lipocalin in G1, G2, G3 and G4

**Fig. 3:**
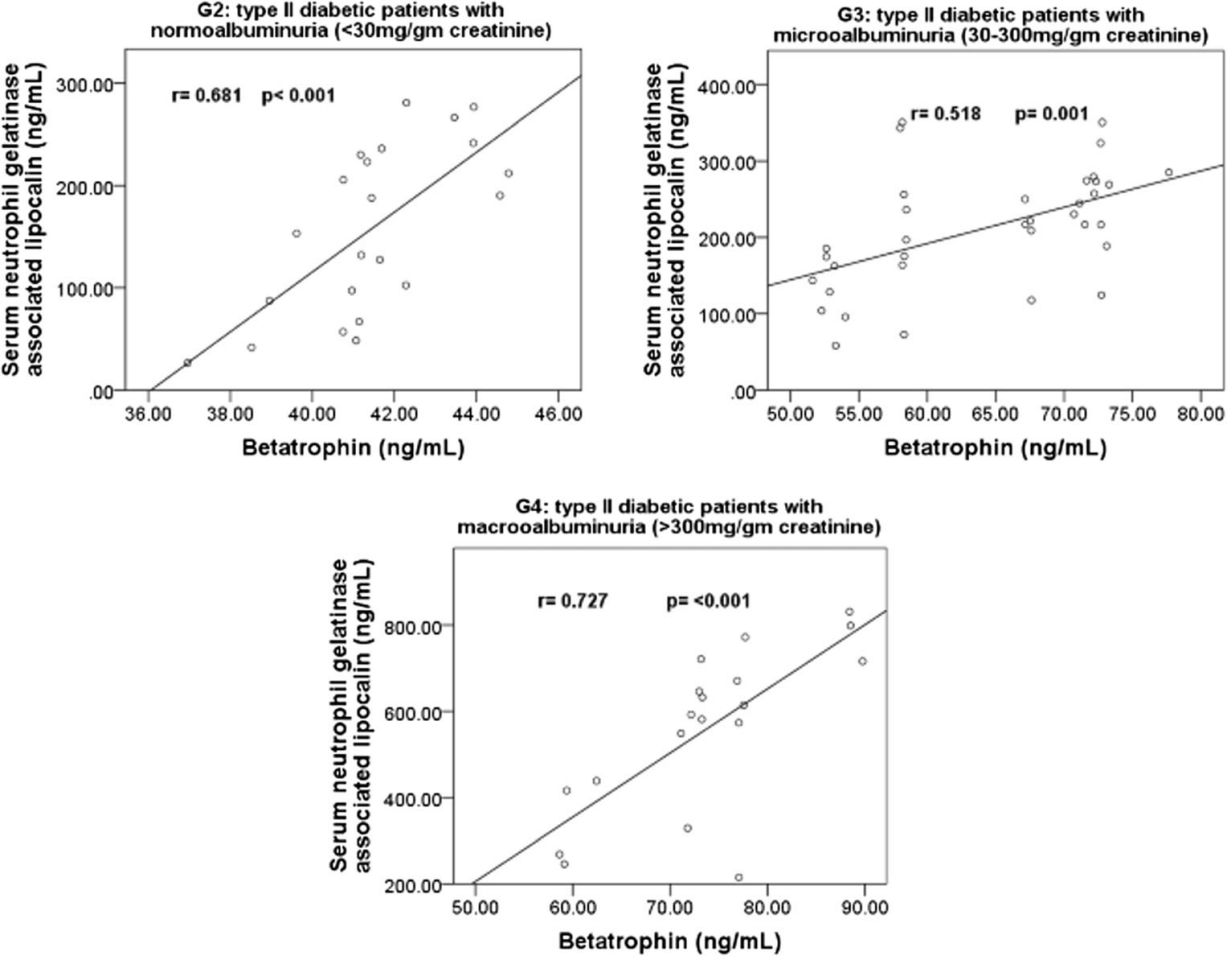
Correlation between serum betatrophin levels and serum neutrophil gelatinase associated lipocalin in G1, G2, G3 and G4

Within the 4 studied groups, the significant correlations between sestrin 2, betatrophin and sNGAL are shown in Figs. 1, 2 and 3**.** Also, within the 4 studied groups, there were significant negative correlations between serum sestrin 2 levels and the BMI in G2 (r = − 0.501, *p* = 0.018), G3 (r = − 0.383, *p* = 0.023) and in G4 (r = − 0.814, *p* < 0.001), between serum sestrin 2 levels and fasting serum blood glucose levels in G3 (r = − 0.501, *p* = 0.002) and in G4 (r = − 0.479, *p* = 0.038), between sestrin 2 levels and serum creatinine levels in G2 (r = − 0.460, *p* = 0.04), G3 (r = − 0.341, *p* = 0.045) and in G4 (r = − 0.467, *p* = 0.044), between sestrin 2 levels and glycated albumin% in G3 (r = − 0.651, *p* > 0.001) and in G4 (r = − 0.685, *p* = 0.001) and between sestrin 2 levels and glycosylated hemoglobin% in G3 (r = − 0.340, *p* = 0.037) and in G4 (r = − 0.510, *p* = 0.026).

On the other hand, significant positive correlations were found between serum betatrophin levels and the BMI in G2 (r = 0.533, *p* = 0.016), G3 (r = 0.371, *p* = 0.028) and in G4 (r = 0.501, *p* = 0.029), between serum betatrophin levels and fasting serum blood glucose levels in G3 (r = 0.507, *p* = 0.002) and in G4 (r = 0.573, *p* = 0.01), between betatrophin levels and serum creatinine levels in G3 (r = 0.411, *p* = 0.014), between betatrophin levels and glycated albumin% in G2 (r = 0.609, *p* = 0.003), G3 (r = 0.655, *p* > 0.001) and in G4 (r = 0.719, *p* = 0.001) and between betatrophin levels and glycosylated hemoglobin% in G3 (r = 0.561, *p* > 0.001) and in G4 (r = 0.737, *p* > 0.001).

The effect strength of different studied parameters on the sNGAL (indicator of DN), is shown in Table 4. Significant effects of serum sestrin levels and serum betatrophin levels were noticed.

**Table 4:** Multiple linear regression analysis in the total sample

Independent variables	Standardized β coefficient (95% CI)	*p* value
Age (years)	0.133(−0.2 to 5.9)	0.062
Gender	−0.018 (−62.8 to 47.9)	0.79
Dur. (years)	0.072 (−2.3 to 5.4)	0.43
BMI (kg/m^2^)	−0.034 (−8.3 to 7.1)	0.71
s-FG (mmol/L)	0.009 (−16.7 to 18.5)	0.92
s–Cr (mg/dL)	0.094 (−62.9 to 226.9)	0.26
s- alb (g/L)	−0.070 (−9.3 to 3.3)	0.35
GA %	0.15 (−2.2 to 16.4)	0.13
HbA1c%	−0.06 (−22.2 to 11.8)	0.54
Serum sestrin2 (ng/mL)	−0.19 (−32.3 to −2.8)	0.032
Serum betatrophin (ng/mL)	0.63 (3.4 to 8.4)	>0.001

Serum neutrophil gelatinase associated lipocalin is the dependent variable.

*Dur* duration of diabetes in years, *BMI* body mass index, *s-FG* serum fasting glucose, *s–Cr* serum creatinine, *s-alb* serum albumin, *GA%* serum glycated albumin%, *HbA1c%* glycosylated hemoglobin%, *CI* confidence interval.

*p*-value ≤ 0.05 is significant correlation

**Fig. 4:**
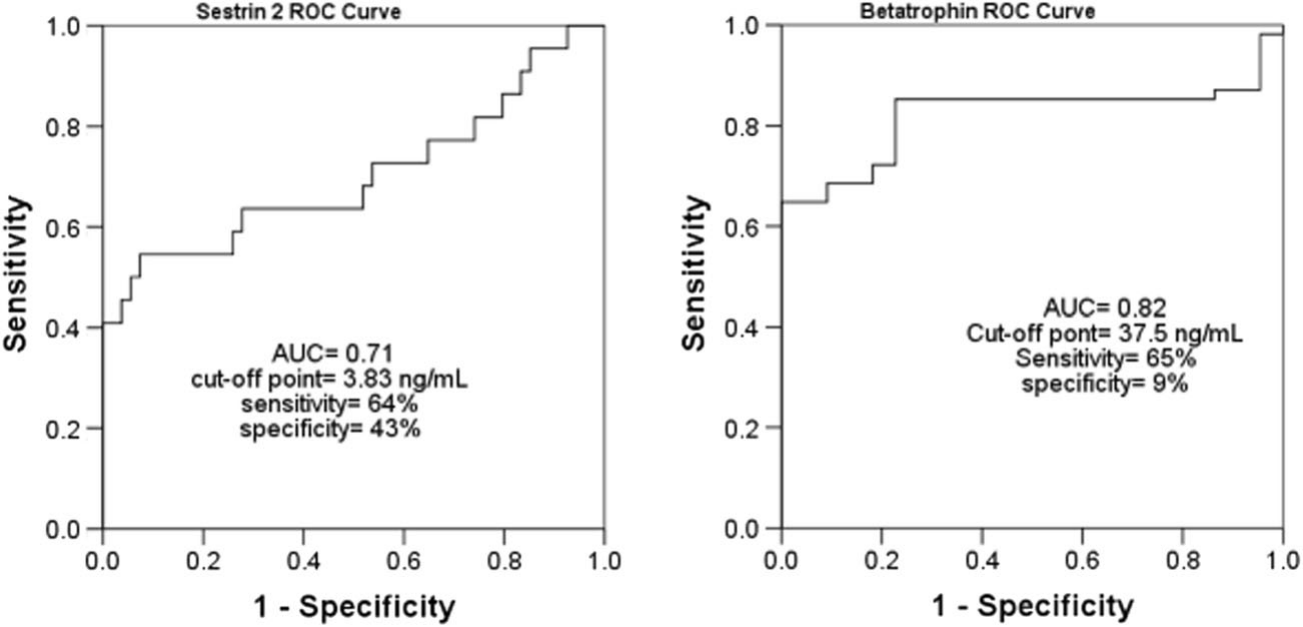
The receiver operating characteristic (ROC) shows the efficacies of serum sestrin 2 levels, serum betatrophin levels in differentiating patient with diabetic nephropathies from diabetic-only individuals. AUC: area under ROC curve

The receiver operating characteristic (ROC) for the efficacies of serum sestrin 2 and betatrophin levels in differentiating patients with diabetic nephropathy from diabetic-only patients is shown in Fig. 4.

## Discussion

The current work is a case-control study that measures the levels of serum sestrin 2 and betatrophin in healthy as well as T2DM patients (with/without DN). Also, it tested the correlation between these levels and the levels of sNGAL (an indicator for DN) [11].

Sestrins are a group of proteins which are inducible by oxidative stress, inflammation and DNA damage. They maintain cellular integrity under stressful conditions through the stimulation of energy producing metabolic reactions and the DNA repair system [12]. Sestrins dampen the action of the free radicals either by their direct action on the anti-oxidant enzymes (peroxiredoxins) or by the regulation of the expression of many anti-oxidant genes (e.g. nuclear factor erythroid factor 2) [13, 31, 32].

Intracellular signaling through the mammalian target of rapamycin complex 1 (mTOR-C1) leads to an increase in cellular proteins and fat synthesis and the development of many adverse metabolic consequences in obese individuals [33]. Chronic activation of mTOR-C1 can lead to insulin resistance by inhibition of the phosphorylation of insulin receptor substrates [34]. Sestrin 2 activates the intracellular AMP-dependent protein kinase (AMPK) enzyme either by a direct binding or by enhancing its gene expression. The activated AMPK inhibits mTOR-C1 [35]. This inactivation of mTOR-C1 by sestrin 2 protects the endoplasmic reticulum against many types of cellular stresses [36] and also decreases the intracellular accumulation of lipids [12]. Moreover, the mTOR-C1 inactivation enhances the action of phosphatidyl-inositol-3-kinase (PI3K) enzyme which promotes the insulin signaling and decreases the insulin resistance and its sequelae [12]. Sestrins also regulates many other cellular functions and metabolic aspects through their effect on the AMPK and mTOR-C1 pathways [14].

The current study found significant low levels of serum sestrin 2 in T2DM patients when compared to the healthy control. Within the T2DM patients’ groups, serum sestrin 2 levels were low in the macroalbuminuricgroup followed by microalbuminuric group then normoalbuminuric group. Also, the present study found significant negative correlations between the levels of serum sestrin 2 and serum betatrophin, NGAL levels. Moreover, significant negative correlations were found between serum sestrin 2 levels and BMI, glycosylated hemoglobin%, serum creatinine, glucose, albumin, glycated albumin%. Measuring serum sestrin 2 levels showed about 71% AUC to differentiate patients with DN from diabetic only cases. The sensitivity was 64% and specificity was 44%. These results go with the results of the study conducted by Nourbakhsh et al. [2017] which revealed low levels of sestrin 2 in obese children when compared to children with normal weight and these levels were negatively correlated with the BMI [17]. Also, Lee et al. [2012] and chai et al. [2015] found that the decrease in the intracellular levels of sestrin 2 led to many adverse consequences such as oxidative stress, mitochondrial dysfunction, insulin resistance and accelerated development of T2DM [14, 15]. On contrary, Chung et al. [2018] found high serum sestrin 2 levels in obese and T2DM patients when compared to the healthy control and these levels correlated positively with the BMI, the levels of serum triacylglycerol, glucose, C-reactive protein and with the degree of insulin resistance [16]. Indeed, the current work couldn’t explain this contradiction.

The expression of betatrophin occurs mainly in live and adipose tissues. It enhances the glucose tolerance and can regulate lipid metabolism [22, 23].The current study foundhigher levels of serum betatrophin in T2DM cases than the healthy control. Within the T2DM patients’ groups, serum betatrophin levels were high in macroalbuminuric group followed by microalbuminuric group the normoalbuminuric group. Also, the present work found a significant positive correlation between serum betatrophin levels and serum NGAL levels. Moreover, significant positive correlations were found between serum betatrophin levels and the BMI, glycosylated hemoglobin%, glycated albumin% and fasting serum blood glucose and creatinine levels. These results are in accordance with the results of many previously conducted studies [24, 27, 28]. Maurer et al. [2017] found significant high levels of betatrophin in T2DM patients and these levels were positively correlated with serum fasting glucose levels and glycosylated hemoglobin % [37]. Chen et al. [2016] reported a significant rise in betatrophin levels in T2DM patients. These levels were higher in macroalbuminuric than normoalbuminuric cases and showed positive correlations with urinary albumin concentration [28]. On the other hand, the results of the present are on contrary to the results of many other studies that which found a decline in the circulating betatrophin levels in T2DM patients [25, 26]. Measuring serum betatrophin levels showed about 82% AUC to differentiate patients with DN from diabetic only cases. The sensitivity was 65% and specificity was 9%.The main limitation of the current study is the small sample size and being case-control. The authors declare that they have no conflict of interest.

## Conclusion

Serum sestrin 2 levels decrease significantly while betatrophin levels increase significantly in T2DM patients with DN especially those with macroalbuminuria. These levels have significant effect strengths on the indicator of diabetic nephropathy; sNGAL which might indicate a critical role of low sestrin 2 levels and high betatrophin levels in the pathogenesis of DN. Drugs that increase the intracellular levels of sestrin 2 may be valuable in the control of T2DM and timely detection and prevention of the development of DN.

## Abbreviations

T2DM: type 2 diabetes mellitus
DN: diabetic nephropathy
sNGAL: serum neutrophil gelatinase associated lipocalin
KDa: kilodalton
ACR: urinary albumn to creatinine ratio
EDTA: ethylendiaminetetraacetic acid
ELISA: enzyme-linked immunosorbant assay
SPSS: statistical package for social sciences
ANOVA: analysis of variance
AMP: adenosine monophosphate
AMPK: AMP-dependent protein kinase
mTOR-C1: mammalian target of rapamycin complex 1
Dur: duration of diabetes in years
BMI: body mass index
s-FG: serum fasting glucose
s–Cr: serum creatinine
s-alb: serum albumin
GA%: serum glycated albumin%
HbA1c%: glycosylated hemoglobin%.

## References

[1] Mohan H (2005). Textbook of pathology.

[2] World Health Organization (WHO). Global report on diabetes. WHO Library Cataloguing-in-Publication Data. 2016; ISBN 978 92 4 156525 7. Available at http://who.int.

[3] American Diabetes Association (2009). Diagnosis and classification of diabetes mellitus. Diabetes Care.

[4] Ma Z, Chen R, Ren H, Guo X, Chen J, Chen L (2014). Endothelial nitric oxide synthase (eNOS) 4b/a polymorphism and the risk of diabetic nephropathy in type 2 diabetes mellitus: a meta-analysis. Meta Gene.

[5] Gariani K, de Seigneux S, Pechère-Bertschi A, Philippe J, Martin PY (2012). Diabetic nephropathy: an update. Rev Med Suisse.

[6] Obineche EN, Adem A (2005). Update in diabetic nephropathy. Int J Diabetes Metab.

[7] Bangstad HJ, Seljeflot I, Berg TJ, Hanssen KF (2009). Renal tubulointerstitial expansion is associated with endothelial dysfunction and inflammation in type 1 diabetes. Scand J Clin Lab Invest.

[8] Caramori M, Fioretto P, Mauer M (2006). Enhancing the predictive value of urinary albumin for diabetic nephropathy. J Am Soc Nephrol.

[9] Ellulu MS, Patimah I, Khaza’ai H, Rahmat A, Abed Y (2017). Obesity and inflammation: the linking mechanism and the complications. Arch Med Sci.

[10] Donath MY, Shoelson SE (2011). Type 2 diabetes as an inflammatory disease. Nat Rev Immunol.

[11] Wolowczuk I (2015). Obesity–an inflammatory state. Acta Vet Scand.

[12] Lee JH, Budanov AV, Karin M (2013). Sestrins orchestrate cellular metabolism to attenuate aging. Cell Metab.

[13] Shin BY, Jin SH, Cho IJ, Ki SH (2012). Nrf2-ARE pathway regulates induction of Sestrin-2 expression. Free Radic Biol Med.

[14] Lee JH, Budanov AV, Talukdar S, Park EJ, Park HL, Park HW, Bandyopadhyay G, Li N, Aghajan M, Jang I, Wolfe AM, Perkins GA, Ellisman MH, Bier E, Scadeng M, Foretz M, Viollet B, Olefsky J, Karin M (2012). Maintenance of metabolic homeostasis by Sestrin2 and Sestrin3. Cell Metab.

[15] Chai D, Wang G, Zhou Z, Yang H, Yu Z. Insulin increases sestrin 2 content by reducing Its degradation through the PI3K/mTOR signaling pathway. Int J Endocrinol. 2015, Article ID 505849, 9 pages. 10.1155/2015/505849 PMC4352509 25792980

[16] Chung HS, Hwang HJ, Hwang SY, Kim NH, Seo JA, Kim SG, Kim NH, Baik SH, Choi KM, Yoo HJ (2018). Association of serum Sestrin2 level with metabolic risk factors in newly diagnosed drug-naïve type 2 diabetes. Diabetes Res Clin Pract.

[17] Nourbakhsh M, Sharifi R, Ghorbanhosseini SS, Javad A, Ahmadpour F, Razzaghy Azar M, Larijani B (2017). Evaluation of plasma TRB3 and Sestrin 2 levels in obese and normal-weight children. Child Obes.

[18] Bolignano D, Donato V, Coppolino G, Campo S, Buemi A, Lacquaniti A, Buemi M (2008). Neutrophil gelatinase–associated lipocalin (NGAL) as a marker of kidney damage. Am J Kidney Dis.

[19] Devireddy LR, Gazin C, Zhu X, Green MR (2005). A cell surface receptor for lipocalin 24p3 selectively mediates apoptosis and iron uptake. Cell..

[20] Bolignano D, Lacquantiti A, Coppolino G, Donato V, Fazio MR, Nicocia G, Buemi MG (2009). Neutrophil gelatinase-associated Lipocalin as an early biomarker of nephropathy in diabetic patients. Kidney Blood Press Res.

[21] Lacquaniti A, Donato V, Pintaudi B, Di Vieste G, Chirico V, Buemi A, Di Benedetto A, Arena A, Buemi M (2013). “Normoalbuminuric” diabetic nephropathy: tubular damage and NGAL. Acta Diabetol.

[22] Yi P, Park JS, Melton DA (2013). Betatrophin: a hormone that controls pancreatic beta cell proliferation. Cell..

[23] Wang Y, Quagliarini F, Gusarova V, Gromada J, Valenzuela DM, Cohen JC, Hobbs HH (2013). Mice lacking ANGPTL8 (betatrophin) manifest disrupted triglyceride metabolism without impaired glucose homeostasis. Proc Natl Acad Sci U S A.

[24] Fu Z, Berhane F, Fite A, Seyoum B, Abou-Samra AB, Zhang R (2014). Elevated circulating lipasin/betatrophin in human type 2 diabetes and obesity. Sci Rep.

[25] Gomez-Ambrosi J, Pascual E, Catalan V, Rodriguez A, Ramirez B, Silva C (2014). Circulating betatrophin concentrations are decreased in human obesity and type 2 diabetes. J Clin Endocrinol Metab.

[26] Al-Rawashdeh A, Kasabri V, Bulatova N, Akour A, Zayed A, Momani M, Khawaja N, Bustanji H, Hyasat D (2017). The correlation between plasma levels of oxytocin and betatrophin in non-diabetic and diabetic metabolic syndrome patients: A cross sectional study from Jordan. Diabetes Metab Syndr: Clinical Research and Reviews.

[27] Espes D, Martinell M, Carlsson PO. Increased circulating betatrophin concentrations in patients with type 2 diabetes. Int J Endocrinol. 2014;2014. 10.1155/2014/323407. 10.1155/2014/323407 PMC4055101 24963292

[28] Chen CC, Susanto H, Chuang WH, Liu TY, Wang CH (2016). Higher serum betatrophin level in type2 diabetes subjects is associated with urinary albumin excretion and renal function. Cardiovasc Diabetol.

[29] Rodby RA, Rohde RD, Sharon Z, Pohl MA, Bain RP, Lewis EJ (1995). The urine protein to creatinine ratio as a predictor of 24-hour urine protein excretion in type 1 diabetic patients with nephropathy. The collaborative study group. Am J Kidney Dis.

[30] Parchwani DN, Upadhyah AA (2012). Diabetic nephropathy: progression and pathophysiology. Int J Med Sci Public Health.

[31] Budanov AV (2011). Stress-responsive sestrins link p53 with redox regulation and mammalian target of rapamycin signaling. Antioxid Redox Signal.

[32] Budanov AV, Sablina AA, Feinstein E, Koonin EV, Chumakov PM (2004). Regeneration of peroxiredoxins by p53-regulated sestrins, homologs of bacterial AhpD. Science.

[33] Wullschleger S, Loewith R, Hall MN (2006). TOR signaling in growth and metabolism. Cell..

[34] Howell JJ, Ricoult SJ, Ben-Sahra I, Manning BD (2013). A growing role for mTOR in promoting anabolic metabolism. Biochem Soc Trans.

[35] Eid AA, Lee DY, Roman LJ, Khazim K, Gorin Y (2013). Sestrin 2 and AMPK connect hyperglycemia to Nox4-dependent endothelial nitric oxide synthase uncoupling and matrix protein expression. Mol Cell Biol.

[36] Ro SH, Xue X, Ramakrishnan SK, Ck C, Namkoong S, Jang I (2016). Tumor suppressive role of sestrin2 during colitis and colon carcinogenesis. eLife.

[37] Maurer L, Schwarz F, Fischer-Rosinsky A, Schlueter A, Brachs S, Mo M, et al. Renal function is independently associated with circulating betatrophin. PLoS One. 2017;12(3). 10.1371/journal.pone.0173197 PMC5336269 28257453

